# Mechanisms of the Beneficial Actions of Ischemic Preconditioning on Subcellular Remodeling in Ischemic-Reperfused Heart

**DOI:** 10.2174/157340310793566118

**Published:** 2010-11

**Authors:** By Alison L Müller, Naranjan S Dhalla

**Affiliations:** Institute of Cardiovascular Sciences, St Boniface Hospital Research Centre, and Department of Physiology, Faculty of Medicine, University of Manitoba, Winnipeg, Manitoba, Canada R2H 2A6

**Keywords:** Cardioprotection, ischemia-reperfusion injury, ischemic preconditioning, oxidative stress, reactive oxygen species, subcellular organelles.

## Abstract

Cardiac function is compromised by oxidative stress which occurs upon exposing the heart to ischemia reperfusion (I/R) for a prolonged period. The reactive oxygen species (ROS) that are generated during I/R incur extensive damage to the myocardium and result in subcellular organelle remodeling. The cardiac nucleus, glycocalyx, myofilaments, sarcoplasmic reticulum, sarcolemma, and mitochondria are affected by ROS during I/R injury. On the other hand, brief periods of ischemia followed by reperfusion, or ischemic preconditioning (IPC), have been shown to be cardioprotective against oxidative stress by attenuating the cellular damage and alterations of subcellular organelles caused by subsequent I/R injury. Endogenous defense mechanisms, such as antioxidant enzymes and heat shock proteins, are activated by IPC and thus prevent damage caused by oxidative stress. Although these cardioprotective effects of IPC against I/R injury are considered to be a consequence of changes in the redox state of cardiomyocytes, IPC is considered to promote the production of NO which may protect subcellular organelles from the deleterious actions of oxidative stress. The article is intended to focus on the I/R-induced oxidative damage to subcellular organelles and to highlight the cardioprotective effects of IPC. In addition, the actions of various endogenous cardioprotective interventions are discussed to illustrate that changes in the redox state due to IPC are cardioprotective against I/R injury to the heart.

## INTRODUCTION

Oxidative stress plays a role in a number of cardiovascular diseases including hypertension, cardiomyopathy, cardiac hypertrophy, heart failure, and ischemia-reperfusion injury (I/R) [1, 2]. The I/R phenomenon pertains to cell damage induced by reactive oxygen species (ROS) and is associated with the development of intracellular Ca^2+^ overload as well as deleterious effects on subcellular organelles [1, 2], (see Fig. (**1**)). The increase in intracellular Ca^2+ ^due to I/R injury consequently triggers a variety of chemical reactions that further augment production of ROS [3]. The primary sources of oxidative stress in the heart are mitochondrial cytochromes, as well as xanthine oxidoreductase, NAD(P)H oxidase, and nitric oxide synthase (NOS) [4]. A positive feedback loop concerning “ROS-induced ROS release” has been proposed after observing that ROS associating with mitochondrial depolarization resulted in a further release of ROS from the mitochondria [5]. This release of ROS changes the intracellular environment from its original reducing state to oxidizing milieu signaling causing the eventual expiration of the cell [6]. In particular, during the ischemic phase the level of ROS is relatively low [7], however upon reperfusion of the ischemic heart, bursts of ROS have been observed using electron paramagnetic resonance spectroscopy, spin trap [alpha]-phenyl-*N-tert*-butylnitrone, and luminal-enhanced ter-butyl-initiated chemiluminesence [1]. 

Clinical procedures including angioplasty, thrombolytic therapy, coronary bypass surgery, and cardiac transplantation are considered to cause I/R injury where the ischemic insult is prolonged beyond a certain critical period [1]. It can also occur following the termination of an angina attack [1]. This large burst of ROS following reperfusion can decrease cardiac function by inhibiting contractile activity [8], altering membrane permeability [9], and increasing cell death [10]. The damage caused by I/R injury can potentially be attenuated by cardiac preconditioning, first observed in the canine myocardium [11]. This phenomenon has been extensively studied since its initial detection in 1986 and has been found to be instrumental in delaying myocardial necrosis and reduce cell apoptosis, diminishing I/R-induced ventricular arrhythmias, preserving post-ischemic endothelial function, attenuating neutrophil-mediated inflammation response in the myocardium, and improving contractile function [11, 12]. Ischemic preconditioning (IPC) diminishes the effects of both endogenous and exogenous oxidative stress [13] and limits the cycle of depressive pro-inflammatory cytokine production [14], reducing myocardial infarct size and improving cardiac function. For a summary of possible mechanisms of IPC refer to Table **1**. It should be mentioned that IPC is normally produced by subjecting the heart to three to five cycles of brief ischemia followed by brief reperfusion and is now well known to be cardioprotective against I/R-induced injury. 

## ALTERED FUNCTIONS OF SUBCELLULAR ORGANELLES BY ROS AND IPC

During ischemia there is a transition from aerobic to anaerobic metabolism in the heart that leads to a reduction in energy production depleting readily available high energy phosphate stores including ATP and creatine phosphate [15]. As a result, not only is the cardiac contractile function decreased, but also the function of energy-dependent ion pumps which cause an accumulation of metabolites and cations and leads to acidosis, increases the osmotic load, activates Ca^2+^-dependent enzymes and produces ROS [15]. The components of the myocardial cell, which are modified by ROS and ultimately lead to the overall change in myocardial function, include the nucleus, glycocalyx (extracellular matrix), cardiac myofilaments, sarcoplasmic reticulum (SR), sarcolemma (SL) and mitochondria of the myocardial cell. It is pointed out that excessive amounts of ROS, which are formed during the development of I/R injury, are considered to result in oxidative stress and produce deleterious effects on the myocardium. On the other hand, low concentrations of ROS, which are generated upon subjecting the heart to IPC, result in changing the redox state of cardiomyocytes and produce beneficial actions against I/R injury [12]. 

### Cardiac Nucleus

Modifications of the cardiac nucleus are important in IPC due to the observation that it undergoes changes that contribute to both early phase IPC, occurring 1-3 hours after stimulus, and delayed phase IPC, occurring 12-24 hours after IPC stimulus [16]. Delayed phase IPC is caused by the upregulation of cardioprotective genes that occurs during the initial IPC stimulus which progresses to transcription regulation and then translation [17]. It includes the upregulation of VEGF, for angiogenesis promotion, and the anti-apoptotic Bcl-2 protein family, to attenuate apoptosis. There have also been observed increases in mRNA levels for heat shock proteins (HSP) 27, 70, and 89 as well as antioxidants such as catalase, glutathione peroxidase, and manganese superoxide dismutase (MnSOD) [18]. The transcription factor most extensively studied with regard to I/R injury and IPC is the nuclear factor κ-light-chain enhancer of activated B cells (NFκB). Elevated mRNA expression and DNA binding of NFκB has been demonstrated during IPC where, initially its translocation to the nucleus is increased, but then after prolonged I/R its activation is reduced [17, 19]. This has an effect on late-phase IPC by producing nitric oxide (NO) *via* inducible nitric oxide synthase (iNOS) upregulation [12] which leads to cyclooxygenase-2 activation and the production of cytoprotective prostaglandins PGE_2_ and PGI_2_, as well as MnSOD and the expression of the anti-apoptotic Bcl-2 gene family [20-22]. Observations involving Bcl-2-associated anthanogene-1 (BAG-1) illustrate interactions with heat shock proteins HSC70 and HSP70 that may promote cell survival. BAG-1 is normally detected in the nucleus, however following ischemia, not only was the expression of both its isoforms increased, it also increased its binding to HSC70 in rat cardiomyocytes [23]. Its expression was not attenuated following I/R, indicating potential cardioprotective properties. In addition, when undergoing IPC, there has been an observed increase in the expression of the fatty acid transport (FAT) gene as well as genes involved with remodeling (fibronectin, laminin, and collagens I and II) [24, 25]. On the other hand, oxidative stress results in the down-regulation of genes corresponding to energy-generating pathways, such as fatty acid metabolism [25]. 

### Cardiac Glycocalyx & Myofilaments

Oxidative stress and IPC have measurable effects on glycocalyx activities and cardiomyocyte myofilaments. The glycocalyx is particularly susceptible to I/R stress as it is the first to be exposed to injury, although preconditioning has also been partially effective in protecting it [26]. IPC reduces the cleavage of myofilament troponin I by matrix metalloprotease 2 (MMP-2) [27] by decreasing its release and activation. In isolated rat hearts, the activation and release of MMP-2 has shown direct correlation with cardiac dysfunction observed in I/R injury [27, 28]. Oxidative stress, in particular stress induced by hydroxyl radicals and peroxynitrite, appear to cause damage in myofibrils, previously noted in chronic atrial fibrillation patients [29], although it may also cause a similar effect as a result of I/R injury. The cytoskeleton has also demonstrated responsiveness in IPC *via* ROS activation of p38 MAPK, which subsequently activates HSP 27. Not only does this cause the polymerization of actin filaments, it also increases the stability of the contractile apparatus through late-phase preconditioning [30-32]. 

### Sarcoplasmic Reticulum

The SR is a key regulator of intracellular concentration of Ca^2+^ in cardiomyocytes. It plays an integral role in maintaining cardiac contractile function as a result of its regulation of Ca^2+^. During I/R injury, it has been observed that cytosolic Ca^2+^ increases dramatically during initial ischemia causing damage to the cell and decreasing ATP [33-35]. Specifically, it has been noted that, even after brief episodes of ischemia, there are changes in SR Ca^2+^ channels [36]. There have also been observed decreases in mRNA levels of SR Ca^2+^-cycling proteins; however, IPC has been demonstrated to have the capacity to attenuate these changes by preventing intracellular Ca^2+^ overload, therefore upholding normal SR function [37, 38]. The ROS species H_2_O_2_ has exhibited direct effects on SERCA (sarcoplasmic reticulum Ca^2+^-stimulated-ATPase) by decreasing its activity and causing NCX (Na^+^-Ca^2+^ exchanger) to increase its activity [39]. This implies that redox-dependent SR Ca^2+^ depletion may be partially affected by this reciprocal regulation of SERCA and NCX in rat ventricular myocytes [39]. Specifically, FeSO_4_/EDTA-induced oxidative stress has shown modification of –SH groups, lysine, tryptophan, and tyrosine which are crucial to optimal Ca^2+^-stimulated-ATPase activity [40]. On the other hand, oxidation of the –SH groups of SR ryanodine receptors (RyRs) have been implicated as being essential in the IPC process for attenuating subsequent prolonged I/R-induced intracellular Ca^2+^-overload [41]. IPC induced cardioprotection of the SR involves mediating Ca^2+^ efflux and influx between the SR and the cytoplasm of cardiomyocytes in order to prevent contractile dysfunction. Understanding the differences between oxidative stress induced by I/R injury from the beneficial effects induced by IPC on the SR could be key in deciphering what signaling is required to cause cardioprotection. 

### Sarcolemma

The phospholipid bilayer boundary of cardiomyocytes, the SL, contains a variety of receptors, as well as cation pumps, exchangers, and channels that are immediately susceptible to redox modification which potentially affects downstream signaling in the cardiomyocyte causing alteration of its normal function. H_2_O_2_ has been specifically noted to have a bi-phasic effect on ATP-binding where the initial increase and then subsequent decrease of binding of ATP occurs in a time and concentration-dependent manner [42]. In addition, HOCl, a well known oxidant, was shown to inhibit ATP-binding; both these observations were found in SL membranes isolated from rat ventricular cardiomyocytes [42]. When porcine heart SL was treated with the xanthine-xanthine oxidase, an oxyradical generating system, a decrease in Na^+^-K^+^-ATPase activity occurred which correlated partially with a decrease in its affinity. It was postulated that the inhibitory effect may be partially due to superoxide radical generation as singlet oxygen was found to decrease cardiac sarcolemmal Na^+^-K^+^-ATPase activity [43, 44]. An interesting study by Fuller *et al.* [45] identified an endogenous, stable inhibitor of cardiac-specific Na^+^-K^+^-ATPase that accumulates in the cell during ischemia in rat hearts. It was found that SL Na^+^-K^+^-ATPase activity rose when SL membranes were purified away from the cytosol, where the inhibitor is located. Production of this inhibitor corresponded with oxidative stress induced by ischemia, indicating that oxidative stress has the capacity to inhibit Na^+^-K^+^-ATPase through this mechanism [43, 45, 46]. The activation of Na^+^-K^+^-ATPase increases levels of Na^+^, observed during ischemia, which can be partially explained by the presence of this inhibitor. Interestingly, the inhibitor was unable to be detected in the cardiac effluent post-reperfusion which the authors postulated could be due to it remaining accumulated in the cytosol of the cell, or it may be inactivated immediately post-reperfusion [45]. It has also been observed that oxygen free radicals can physically disrupt the SL membrane, causing the intracellular space to be exposed to the extracellular environment [9]. In addition, oxidative stress, caused by xanthine-xanthine oxidase, altered the activity of SL phospholipase C which ultimately affects downstream signaling, including the activation of PKC [47]. Furthermore, the activity of SL phospholipase D, which primarily is a part of the signal transduction mechanism for regulating Ca^2+^ movements in the heart, was impaired by I/R injury *via* H_2_O_2_ and HOCl by modification of its functionally critical thiol groups [48]. Also, the thiol groups associated with phosphatidylinositol 4-kinase and phosphatidylinositol 4-P-5 kinase, required for the proper function of numerous SL proteins, were impaired by ROS therefore reducing their function [49]. 

Despite the previously described ROS-induced alterations of SL proteins, the one most discussed cardioprotective effect of preconditioning is the SL version of K^+^-ATP channels (sarc K^+^-ATP channels) and its comparison with mitochondrial K^+^-ATP channels (mito K^+^-ATP channels). These K^+^-ATP channel proteins are activated by NO either from NO synthase (NOS) or extracellularly available NO [15]. Although the general consensus is that mito K^+^-ATP channels play a more significant role regarding the beneficial effects of IPC, sarc K^+^-ATP channels are important because of an increased vulnerability of these sites to oxidative stress [50]. Sarc K^+^-ATP channels were initially noticed as being involved in IPC as they are opened when exposed to free radicals and have been observed to possess cytoprotective properties [51-54]. In a study investigating the difference between sarc and mito K^+^-ATP channels, sarc K^+^-ATP channels appeared to act as an effecter of preconditioning and were found to be important in improving functional recovery. This was indicated when cardioprotective effects induced by sarc K^+^-ATP channels occurred during the stress period, however its activation was not required during the preconditioning period. In freshly isolated adult rat cardiomyocytes, using an isoflurane-induced protection technique to study the effects of preconditioning [55], both sarc and mito K^+^-ATP channels were observed to be required for cardioprotection against oxidative stress. In a specific mouse model knock-out of Kir 6.2 (the pore subunit of sarc K^+^-ATP channels) cardioprotective effects induced by IPC were extirpated, indicating their necessity for cardioprotection in this model [56]. Finally, it was shown that activated sarc K^+^-ATP channels are important in preventing cardiomyocyte apoptosis and mitochondrial damage during stress, as inhibition of these ATP channels promoted the mitochondrial death pathway by augmenting oxidative stress-induced apoptosis. In addition, it was noted that mitochondrial Ca^2+^ loading was also significantly increased upon inhibition of the sarc K^+^-ATP channel in cultured HL-1 and neonatal cardiomyocytes [57]. Clearly, the oxidative stress-induced alterations on the SL play a significant role in IPC, and despite the discussions comparing the significance between sarc and mito K^+^-ATP channels, it is clear that, not only do the sarc K^+^-ATP channels play an important role in IPC, but other proteins of the SL do as well. 

### Mitochondria

There is an ample amount of literature discussing how mitochondria are involved in IPC. Mitochondria maintain the balance between cell life and cell apoptosis, where the key to its activity is its generation of ROS and free radicals within the cell. It appears that the majority of pathways currently discussed to be effective in cardioprotection converge on the mitochondrial permeability pore in an effort to keep it closed; specifically to preserve its inner membrane potential and reduce mitochondrial Ca^2+^ to ensure uninterrupted energy production [15]. In the previously discussed study comparing sarc K^+^-ATP and mito K^+^-ATP channels [55], mito K^+^-ATP channels acted as both an effecter of IPC and its trigger. Mito K^+^-ATP channels were observed to be activated during the preconditioning period and during the exposure to oxidative stress. In contrast to the sarc K^+^-ATP channels, where they were found to be important for improving functional recovery, mito K^+^-ATP channels were shown to have an effect primarily on infarct size [55]. The pharmacological preconditioning of the rat heart, using diazoxide, has shown to cause the opening of mito K^+^-ATP channels and protect against ischemia-induced ventricular arrhythmias [58]. It was also demonstrated that in the mitochondrial protective pathway, the ROS released from the mitochondria during IPC caused the opening of mito K^+^-ATP channels upstream and was protective [59]. An extensive study carried out by Das and Sarker [60] on mito K^+^-ATP channels by using nicorandil or minoxidil, found that mito K^+^-ATP channels have the potential to protect against I/R-induced arrhythmias, reduce myocardial infarct size, and increase cardiomyocyte survival in the intact anesthetized rabbit heart. The mito K^+^-ATP channels have been observed to have properties that detect local oxidants resulting in decreased free radical generation. These sensors are likely located in the channel’s sulfonylurea receptor where stimulation by reactive oxygen prevents mitochondrial ROS release [61]. Although some variations among the results are evident in different animal models, as well as different means of inducing IPC and/or I/R injury, it is clear that mito K^+^-ATP channels play some role in IPC in protecting the heart against I/R injury.

The functions of mitochondria have been shown to be altered upon exposure of the heart to IPC including the expression of the mitochondrial antioxidant, manganese superoxide dismutase (MnSOD), *via* the formation of NFκB and activator protein-1 (AP-1) [21]. There is also increased ATP production in preconditioned hearts when compared to non-preconditioned hearts [62]. The phenomenon of “ROS-induced ROS release” involves opening of the mitochondrial permeability transition pore (mPTP) by ROS to release additional ROS, validating the mitochondria as the primary source of ROS in the cell [5, 10]. Interestingly, IPC prevents the mPTP from opening so the burst of ROS does not occur which leads to an overall reduction of oxidative stress in the cell [5]. An intriguing review on the inhibitory effect of IPC on mitochondrial respiratory complexes has discussed how IPC could cause gradual activation of mitochondrial function; ultimately bypassing ROS bursts and Ca^2+^ overload [63]. Complex I (NADH ubiquinone oxidoreductase) represents the electron entry into the mitochondria and is a major site of ROS generation [64, 65]. The regulation of NADH/NAD^+^ redox balance was shown to influence mPTP opening where an increase in NADH/NAD^+^ ratio inhibited its opening [63, 66]. IPC and NO also inhibited the activity of Complex I thus minimizing ROS generation [63]. Complex I was reversibly inhibited by S-nitrosation, a potential mechanism for NO-dependent mitochondria respiratory chain control [67]; inhibition of this complex by as little as 25% has demonstrated significant inhibition of the respiratory chain, as it is the entry point for electrons [67, 68]. Complex II (succinate dehydrogenase) has been connected to mito K^+^-ATP channel function where inhibition of complex II was found to open mito K^+^-ATP channels and result in cardioprotection [63, 69]. Complex III (cytochrome bc1 complex) is another site of ROS formation in the electron transport chain, where its inhibition is a function of its own ROS generation [63]. Unfortunately, how this complex is affected by IPC is currently unknown. Complex IV (cytochrome c oxidase) has been shown to be inhibited by NO∙, which is released during I/R [63, 70]. Glyceraldehyde 3-phosphate dehydrogenase (GAPDH) was also inhibited by endogenously derived nitrolipids generated during IPC [71, 72]. This causes the accumulation of fructose-1,6-bisphosphate (F-1,6-BP) which has been observed to improve glycolytic flux and functional recovery in post-ischemic myocardium [73]. In addition, the quantity of lactate, the final product of anaerobic glycolysis that occurs during ischemia, was observed to be nine-fold less in pre-conditioned hearts compared to non-preconditioned hearts [73]. This demonstrates the ability of IPC to inhibit glycolysis resulting in the prevention of acidosis [64]. As a final note, cytosolic to mitochondrial relocation of hexokinase has been shown to occur in IPC, moderating cytochrome *c* release and ROS production [74, 75]. 

## ENDOGENOUS CARDIOPROTECTIVE INTERVENTIONS

A number of proteins and molecules are involved in cardioprotection and are influenced by the redox state induced by IPC. The majority of these proteins had, at some point, been involved following the release of small amounts of ROS. Low NO preconditioning of H9c2 (an embryonal rat heart-derived cell line) cells can induce the production of the cyclooxygenase-2 (COX-2) protein [76]. Although COX-2 is upregulated in oxidative stress-induced injury and apoptosis, it was found to be cardioprotective upon the conversion of arachidonic acid to PGH_2_. It is pointed out that PGH_2_ is further derived into cytoprotective prostanoids, PGE_2_ and PGI_2_, which were found to attenuate stunning and reduce infarct size after I/R [77-80]. PKCε has also been attributed to IPC as it is activated by low concentrations of oxygen radicals and has been observed to be involved in IPC cardioprotection [81-84]. The activation of phospholipase (PL) C and D causes the subsequent release of diacylglycerol, a PKC activator, suggesting PKC as a molecule involved in IPC [15]. PLD has been implied to evoke a cell-survival response upon exposure to low concentrations of oxidants for a brief length of time [85]. In particular, PLD 2 has been noted to have elevated activity when reperfusion occurs briefly after a brief period of ischemic insult to the heart, but its activity is reduced during prolonged reperfusion [86]. Preconditioning has also been illustrated as being promoted by various receptors such as adenosine, adrenergic, bradykinin, and opiod receptors as well as limiting the cycling of depressive pro-inflammatory cytokine production [14, 87]. Thioredoxin, a sulfide reductase, plays a part in maintaining the redox activity inside the cell whose activity is propagated by oxidative stress [88, 89]. It has been shown to be instrumental in transmitting the survival signal in ischemic myocardium [90]; however, it is down-regulated after I/R injury [91]. Interestingly, post-IPC appears to cause an upregulation in thioredoxin, and, in transgenic mouse hearts where there are extra copies of its gene, the cardiomyocytes were resistant to apoptosis [91].

Cardiomyocytes have a few antioxidants that are able to protect the cell from oxidative stress. It is important to protect the cell from oxidative stress, both intracellularly and extracellularly, and the first endogenous antioxidant to encounter ROS is extracellular superoxide dismutase (E-SOD). There are also two additional isozymes, the copper/zinc-containing SOD (CuZn-SOD) localized in the cytosol and the previously described Mn-SOD, present in the mitochondria [92]. These scavenging proteins have also been found to be upregulated after delayed preconditioning [18, 93]. The effects of SOD in cardioprotection are slightly controversial as it has been shown that neither IPC nor I/R affect its activity in cardiomyocytes [94]. It has been suggested that antioxidant activities may only be altered by episodes of intense myocardial I/R injury [94]. Extracellularly, IPC causes the activation of a SOD-like anti-O_2_∙ mechanism reducing the oxyradical burst [94], and protecting the glycocalyx [26]. Despite the uncertainty regarding the beneficial effect of IPC on SOD levels, other antioxidants have been observed to be upregulated during IPC. This includes mitochondrial uncoupling proteins (UCPs) 2 and 3, where their upregulation is inversely proportional to infarct size in preconditioned hearts [95]. The preconditioned mitochondria were found to produce less hydrogen peroxide compared to control. These UCPs are activated by ROS signaling and are thought to protect the cell from excessive ROS generation in an automatic regulatory forward way [96, 97] which would explain their effectiveness in reducing infarct size in preconditioned hearts. Glutathione (GSH) is an intracellular antioxidant that scavenges ∙OH, HOCl, peroxynitrate and O_2_∙ radicals during times of oxidative stress [98]. IPC has been found to preserve the levels of GSH in isolated rabbit hearts [13, 98] which partially explains the reduced amount of oxidative damage sustained by preconditioned hearts. Vitamin C, also known as ascorbic acid, is another endogenous antioxidant that, not only has been shown to react with ROS *in vivo*, but is able to remain in cells for long periods [99, 100]. Vitamin C has the ability to protect against H_2_O_2_-induced cell injury in H9c2 cells [99] and has been shown to be surprisingly effective in protecting plasma lipoproteins from aqueous peroxy radical damage [101]. There is still some debate as to how, or even if, antioxidants play a role in IPC, as literature indicates increases [102], decreases [103, 104], or lack of changes [105, 106], in antioxidant levels. However, it is important to keep in mind that endogenous antioxidants are present in myocardial cells and varying experimental protocols involving different species, methods of inducing IPC and/or I/R injury, and the specific antioxidants studied make this area of research still a mystery waiting to be solved. 

## ROS CAUSES CARDIOPROTECTION

Although ROS were originally viewed as having only detrimental impacts upon cellular function, there is now evidence that certain levels and types of ROS may be beneficial and possibly contribute to cardioprotection in IPC myocardium. When considering free radicals, there has been an observed “radical threshold” where, below this threshold, cardiac function is able to recover, however above this threshold recovery is not possible [107]. The catecholamine adrenaline (ADR) is known to evoke a pro-oxidant signal which causes the translocation of protective transcription factors HSF-1 and NFκB. Interestingly, in isolated rat cardiomyocytes, the pro-oxidant signal from ADR also decreases proteosome activity, which can be recovered upon the addition of the ROS scavenger, tiron [108]. It has also been reported that an increase in endogenous ascorbyl free radical formation may improve functional recovery despite its contribution to oxidative stress [109]. 

### Hyperoxia

The utilization of new technology, such as electron paramagnetic resonance (EPR) paired with oxygen sensitive probes, such as LiPc, allow scientists to measure specific PO_2_/redox status *in vivo* [110], and Doppler flow measurement allows for the investigation of changes in oxygen consumption and tissue oxygenation *in vivo* [111]. After I/R, myocardial tissue reveals hyperoxygenation [112] suggesting the possibility that I/R causes the myocardium to utilize less oxygen [111]. It has been shown that ROS and reactive nitrogen species (RNS), formed during I/R, inhibited mitochondrial oxygen consumption [112], however, IPC attenuated hyperoxygenation due to observed higher blood flow and lower PO_2_ and the possible preservation of higher levels of O_2_ utilization [111]. I/R injury was also shown to occur in the brain where the treatment of normobaric hyperoxia (95% O_2_ with 5% CO_2_) immediately after I/R was found to reduce the infarction volume and improve neurological function close to pre-ischemic levels [113]. It was hypothesized that the addition of O_2_ may decrease ROS generation during ischemia, as there was no increase in oxidative stress when normobaric hyperoxia treatment was applied during focal cerebral I/R [113, 114]. Hyperoxia treatment has also been studied in rat hearts [115, 116]. In one study, mechanically ventilated rats were exposed to hyperoxia for 30 minutes before the hearts were isolated and subjected to 30 min of ischemia followed by 2 hr of reperfusion. Hyperoxia was found to improve cardiovascular function, left ventricular end-diastolic pressure, left ventricular end-developed pressure, and reduce infarct size [115]. Another study reported similar results when inducing hyperoxic preconditioning in addition to reductions in cytochrome-c release and DNA fragmentation which suggest that hyperoxia increases cardiomyocyte tolerance by ROS activation of NFκB [116]. In an open-chest rabbit model of I/R, the exposure of the heart to 100% oxygen during ischemia-only, reperfusion-only, and I/R resulted in smaller infarct sizes as well [117]. It is thus evident that low concentrations of ROS play a role in IPC that allow for cardioprotection where alterations in the subcellular organelles may not occur to the point where drastic changes occur that impair cardiac function. 

### Nitric Oxide and Nitric Oxide Synthases

Endogenous NO and NOS - originally thought of as sources of oxidative stress on cardiomyocytes - have properties that contribute to IPC and cardioprotection. The excess O_2_-∙ caused by I/R injury is scavenged by NO donors, thus protecting the myocardium from I/R injury [118, 119]. In newborn rat cardiomyocytes, treatment with an NO donor (sodium nitroprusside) can activate SERCA2a causing an increase in Ca^2+^ uptake and subsequently preventing cytosolic Ca^2+^ upload [120]. NO can also contribute to the S-nitrosation whose primary target is Complex I of the electron transport chain in the mitochondria, causing its decrease in activity which occurs exclusively in I/R [121]. The modification of cysteine residues by NO∙ has been hypothesized to form a “molecular cap” that prevents further oxidation of thiols by ROS, resulting in the regulation of ROS formation from mitochondria and caspase-3 apoptotic activity [122-124]. NOS has been implicated in preconditioning and cardioprotection, as it was observed to decrease infarct size and increase the recovery of left ventricular diastolic pressure; there was a four-fold increase of Hsp90 association with eNOS and increases in eNOS itself in the myocardium [125]. eNOS has demonstrated possessing cardioprotective properties and its involvement in preconditioning has been shown, in a variety of studies, to recruit endothelial progenitor cells (EPC) that express potentially cardioprotective cytokines including NOS isoforms [126]. The complex formed between eNOS and HSP90 allows for phosphorylation enhancement of eNOS *via* Akt, amplifies NO release, and increases production of cyclic guanosine monosphophate [127-129]. Protection has been implied as being partially dependent on this HSP90/eNOS association enhancement and the resulting NO release [130]. IPC has been shown to preserve the function eNOS as well, hastening endothelial recovery and function [131]. 

## CONCLUSION

In I/R, oxidative stress leads to cardiac dysfunction which can be attenuated by IPC. ROS, the cause of oxidative stress, alter the functions of various subcellular organelles in both I/R injury and IPC [12]. The nucleus is shown to upregulate a slew of cardioprotective genes that have shown effects in late-phase IPC [17, 25], largely as a result of increased NFκB action [17]. IPC reduces the effects of MMP-2 ultimately protecting the glycocalyx and increases the stability of contractile myofilaments [27, 30-32]. SR, the principal Ca^2+^ regulator of the cell responsible for maintaining contractile function, undergoes alterations of its Ca^2+^ channels, including SERCA, NCX, and RyRs [36, 39, 41] which are modified in I/R injury and preserved by IPC. Oxidative stress has also shown to have an inhibitory effect on SL Na^+^-K^+^-ATPase [43-45] and to physically disrupt the phospholipid bilayer [9]. With regards to IPC, the most effective protection of the SL is the activation of sarc K^+^-ATP channels, however, the extent of its effect compared to mito K^+^-ATP channels is still a debatable topic. Both these K^+^-ATP channels have been shown to be effective in different animal models where the mitochondrial isoform acts as an effecter and a trigger, and the SL isoform is an effecter [55]. The importance of the SL isoform is emphasized by the studies of Kir 6.2 KO mice where the missing pore subunit of the sarc K^+^-ATP channel prevented protection of I/R injury post-IPC [56]. Mitochondria are the main source of free radicals in the cell, and, coupled with their “ROS-induced ROS release” mechanism, are not only affected by oxidative stress but incur further damage upon the cell [5, 10]. IPC has been shown to inhibit the electron transport chain, particularly at the electron entry point of Complex I [67, 68], suggesting that a low amount of ROS triggers an anti-apoptotic protective measure whereas high amounts of ROS promote mitochondrial release of pro-apoptotic factors for the development of oxidative stress.

Several endogenous protective interventions have also been demonstrated to be activated when triggered by IPC. These include the upregulation of COX-2 [76] and activation of the PKCε pathway [82, 83]. Interestingly, upon low concentrations of oxidant exposure, PLD signals for cell survival are generated during IPC [85]. Thioreduxin, a survival signal transmitter active in ischemic myocardium, also has increased activity in IPC [90]. Despite the existing debate regarding the effectiveness of different antioxidants in IPC, eSOD [26, 92], mitochondrial UCPs 2 and 3 [95], GSH [98], and vitamin C [101] still seem to be involved. Although these antioxidants become more active, there is still need for further exploration on their effects. Oxidative stress itself has demonstrated cardioprotective properties and involvement in IPC. The pro-oxidant signal of ADR increases the translocation of protective transcription factors [108], whereas hyperoxia has also been shown to be protective in both rat and rabbit models of IPC [115-117]. In IPC and cardioprotection NOS, especially eNOS, has shown effectiveness in attenuating oxidative damage, particularly when it forms a complex with HSP90 [125-129]. An outline of proposed pathways differentiating the two phases of preconditioning is displayed in Fig. (**2**). Most of the work on IPC showing beneficial effects for the recovery of cardiac function preceding I/R injury has been carried out in the isolated heart preparation. However, this procedure should be extended to the clinical setting where IPC should be conducted by 3 to 5 cycles of clamping and releasing of the aorta before carrying out coronary bypass surgery, angioplasty, heart transplantation, or thrombolytic therapy. IPC can cause resistance to oxidative damage by altering the redox state of cardiomyocytes and can be protective by increasing the resistance of subcellular organelles to ROS modifications. 

## Figures and Tables

**Fig. (1):**
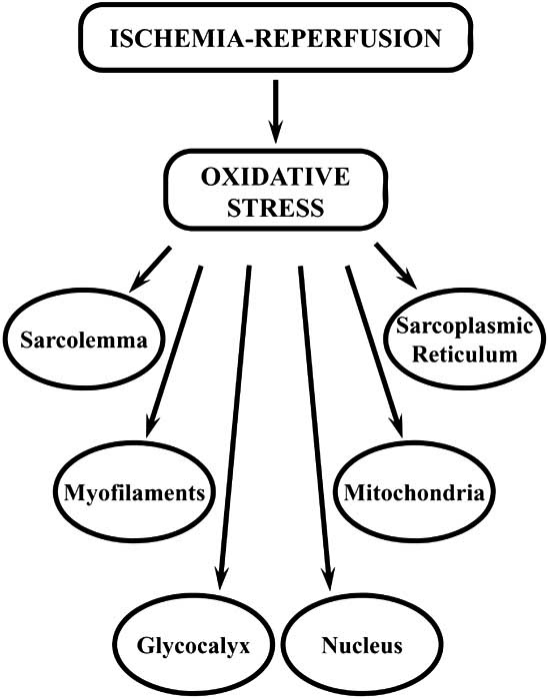
Subcellular organelles affected by oxidative stress induced by ischemia-reperfusion injury.

**Fig. (2):**
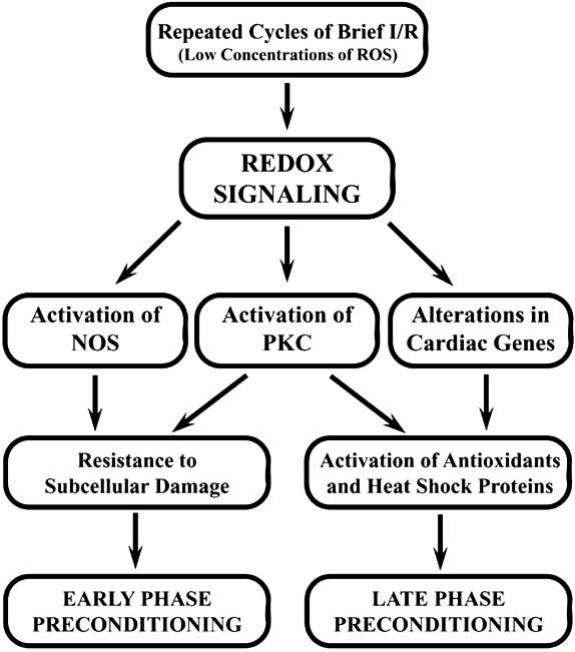
Outline of proposed pathways of redox induced early and late phases of ischemic preconditioning. Abbreviations: I/R, ischemia- reperfusion; ROS, reactive oxygen species; NOS, nitric oxide synthase; PKC, protein kinase C.

**Table 1:** Possible Mechanisms for the Beneficial Effects of Ischemic Preconditioning

1. Low concentrations of ROS and changes in redox state
2. Activation of PKC signaling
3. Formation of adenosine
4. Activation of NOS and production of NO
5. Activation of sarcolemmal K^+^-ATP channels
6. Activation of mitochondrial K^+^-ATP channels
7. Increase in antioxidant reserve
8. Activation of heat shock proteins

Abbreviations: ROS, reactive oxygen species; PKC, protein kinase C; NOS, nitric oxide synthase; NO·, nitric oxide

## References

[1] Dhalla NS, Temsah RM, Netticadan T (2000). Role of oxidative stress in cardiovascular diseases. J Hypertens.

[2] Ytrehus K, Myklebust R, Olsen R, Mjos OD (1987). Ultrastructural changes induced in the isolated rat heart by enzymatically generated oxygen radicals. J Mol Cell Cardiol.

[3] Dhalla NS, Golfman L, Takeda S (1999). Evidence for the role of oxidative stress in acute ischemic heart disease: a brief review. Can J Cardiol.

[4] Penna C, Mancardi D, Rastaldo R, Pagliaro P (2009). Cardioprotection: a radical view - Free radicals in pre and postconditioning. Biochim Biophys Acta.

[5] Zorov DB, Filburn CR, Klotz LO (2000). Reactive oxygen species (ROS)-induced ROS release: a new phenomenon accompanying induction of the mitochondrial permeability transition in cardiac myocytes. J Exp Med.

[6] Das S, Khan N, Mukherjee S (2008). Redox regulation of resveratrol-mediated switching of death signal into survival signal. Free Radic Biol Med.

[7] Vanden Hoek TL, Becker LB, Shao Z, Li C, Schumacker PT (1998). Reactive oxygen species released from mitochondria during brief hypoxia induce preconditioning in cardiomyocytes. J Biol Chem.

[8] Angelos MG, Kutala VK, Torres CA (2006). Hypoxic reperfusion of the ischemic heart and oxygen radical generation. Am J Physiol Heart Circ Physiol.

[9] Arora RC, Hess ML (1985). Effect of reduced oxygen intermediates on sarcolemmal muscarinic receptors from canine heart. Biochem Biophys Res Commun.

[10] Murphy E, Steenbergen C (2008). Mechanisms underlying acute protection from cardiac ischemia-reperfusion injury. Physiol Rev.

[11] Murry CE, Jennings RB, Reimer KA (1986). Preconditioning with ischemia: a delay of lethal cell injury in ischemic myocardium. Circulation.

[12] Saini HK, Machackova J, Dhalla NS (2004). Role of reactive oxygen species in ischemic preconditioning of subcellular organelles in the heart. Antioxid Redox Signal.

[13] Morihira M, Hasebe N, Baljinnyam E (2006). Ischemic preconditioning enhances scavenging activity of reactive oxygen species and diminishes transmural difference of infarct size. Am J Physiol Heart Circ Physiol.

[14] Meldrum DR, Cleveland  JC, Moore EE (1997). Adaptive and maladaptive mechanisms of cellular priming. Ann Surg.

[15] Zaugg M, Schaub MC (2003). Signaling and cellular mechanisms in cardiac protection by ischemic and pharmacological precon-ditioning. J Muscle Res Cell Motil.

[16] Przyklenk K, Kloner RA (1998). Ischemic preconditioning: exploring the paradox. Prog Cardiovasc Dis.

[17] Das DK, Maulik N (2006). Cardiac genomic response following preconditioning stimulus. Cardiovasc Res.

[18] Das DK, Engelman RM, Kimura Y (1993). Molecular adaptation of cellular defences following preconditioning of the heart by repeated ischaemia. Cardiovasc Res.

[19] Morgan EN, Boyle EM, Yun W (1999). An essential role for NF-kappaB in the cardioadaptive response to ischemia. Ann Thorac Surg.

[20] Shinmura K, Xuan YT, Tang XL (2002). Inducible nitric oxide synthase modulates cyclooxygenase-2 activity in the heart of conscious rabbits during the late phase of ischemic preconditioning. Circ Res.

[21] Hoshida S, Yamashita N, Otsu K, Hori M (2002). The importance of manganese superoxide dismutase in delayed preconditioning: involvement of reactive oxygen species and cytokines. Cardiovasc Res.

[22] Maulik N, Engelman RM, Rousou JA (1999). Ischemic preconditioning reduces apoptosis by upregulating anti-death gene Bcl-2. Circulation.

[23] Townsend PA, Cutress RI, Carroll CJ (2004). BAG-1 proteins protect cardiac myocytes from simulated ischemia/reperfusion-induced apoptosis *via* an alternate mechanism of cell survival independent of the proteasome. J Biol Chem.

[24] Maulik N, Das DK (1996). Molecular cloning, sequencing and expression analysis of a fatty acid transport gene in rat heart induced by ischemic preconditioning and oxidative stress. Mol Cell Biochem.

[25] Simkhovich BZ, Marjoram P, Poizat C (2003). Brief episode of ischemia activates protective genetic program in rat heart: a gene chip study. Cardiovasc Res.

[26] Beresewicz A, Czarnowska E, Maczewski M (1998). Ischemic preconditioning and superoxide dismutase protect against endothelial dysfunction and endothelium glycocalyx disruption in the postischemic guinea-pig hearts. Mol Cell Biochem.

[27] Lalu MM, Csonka C, Giricz Z (2002). Preconditioning decreases ischemia/reperfusion-induced release and activation of matrix metalloproteinase-2. Biochem Biophys Res Commun.

[28] Cheung PY, Sawicki G, Wozniak M (2000). Matrix metalloproteinase-2 contributes to ischemia-reperfusion injury in the heart. Circulation.

[29] Rodrigo R, Castillo R, Cereceda M, Asenjo R, Zamorano J, Araya J (2007). Non-hypoxic preconditioning of myocardium against postoperative atrial fibrillation: mechanism based on enhancement of the antioxidant defense system. Med Hypotheses.

[30] Guay J, Lambert H, Gingras-Breton G (1997). Regulation of actin filament dynamics by p38 map kinase-mediated phosphorylation of heat shock protein 27. J Cell Sci.

[31] Landry J, Huot J (1995). Modulation of actin dynamics during stress and physiological stimulation by a signaling pathway involving p38 MAP kinase and heat-shock protein 27. Biochem Cell Biol.

[32] Das DK, Maulik N, Sato M, Ray PS (1999). Reactive oxygen species function as second messenger during ischemic preconditioning of heart. Mol Cell Biochem.

[33] Nakamura T, Hayashi H, Satoh H (1999). A single cell model of myocardial reperfusion injury: changes in intracellular Na^+^ and Ca^2+^ concentrations in guinea pig ventricular myocytes. Mol Cell Biochem.

[34] Nayler WG (1981). The role of calcium in the ischemic myocardium. Am J Pathol.

[35] Nayler WG, Panagiotopoulos S, Elz JS, Daly MJ (1988). Calcium-mediated damage during post-ischaemic reperfusion. J Mol Cell Cardiol.

[36] Temsah RM, Netticadan T, Chapman D (1999). Alterations in sarcoplasmic reticulum function and gene expression in ischemic-reperfused rat heart. Am J Physiol.

[37] Osada M, Netticadan T, Tamura K, Dhalla NS (1998). Modification of ischemia-reperfusion-induced changes in cardiac sarcoplasmic reticulum by preconditioning. Am J Physiol.

[38] Temsah RM, Kawabata K, Chapman D, Dhalla NS (2002). Preconditioning prevents alterations in cardiac SR gene expression due to ischemia-reperfusion. Am J Physiol Heart Circ Physiol.

[39] Kuster GM, Lancel S, Zhang J (2010). Redox-mediated reciprocal regulation of SERCA and Na^+^-Ca^2+^ exchanger contributes to sarcoplasmic reticulum Ca^2+^ depletion in cardiac myocytes. Free Radic Biol Med.

[40] Kaplan P, Babusikova E, Lehotsky J, Dobrota D (2003). Free radical-induced protein modification and inhibition of Ca^2+^-ATPase of cardiac sarcoplasmic reticulum. Mol Cell Biochem.

[41] Zucchi R, Yu G, Galbani P (1998). Sulfhydryl redox state affects susceptibility to ischemia and sarcoplasmic reticulum Ca^2+^ release in rat heart. Implications for ischemic preconditioning. Circ Res.

[42] Musat S, Dhalla NS (1996). Alteration in cardiac sarcolemmal ATP receptors by oxyradicals. Ann N Y Acad Sci.

[43] Shao Q, Matsubara T, Bhatt SK, Dhalla NS (1995). Inhibition of cardiac sarcolemma Na^+^-K^+^ ATPase by oxyradical generating systems. Mol Cell Biochem.

[44] Vinnikova AK, Kukreja RC, Hess ML (1992). Singlet oxygen-induced inhibition of cardiac sarcolemmal Na^+^/K^+^-ATPase. J Mol Cell Cardiol.

[45] Fuller W, Parmar V, Eaton P (2003). Cardiac ischemia causes inhibition of the Na/K ATPase by a labile cytosolic compound whose production is linked to oxidant stress. Cardiovasc Res.

[46] Elmoselhi AB, Butcher A, Samson SE, Grover AK (1994). Free radicals uncouple the sodium pump in pig coronary artery. Am J Physiol.

[47] Meij JT, Suzuki S, Panagia V, Dhalla NS (1994). Oxidative stress modifies the activity of cardiac sarcolemmal phospholipase C. Biochim Biophys Acta.

[48] Dai J, Meij JT, Padua R, Panagia V (1992). Depression of cardiac sarcolemmal phospholipase D activity by oxidant-induced thiol modification. Circ Res.

[49] Mesaeli N, Tappia PS, Suzuki S (2000). Oxidants depress the synthesis of phosphatidylinositol 4,5-bisphosphate in heart sarcolemma. Arch Biochem Biophys.

[50] Slezak J, Tribulova N, Pristacova J (1995). Hydrogen peroxide changes in ischemic and reperfused heart. Cytochemistry and biochemical and X-ray microanalysis. Am J Pathol.

[51] Gross GJ, Auchampach JA (1992). Blockade of ATP-sensitive potassium channels prevents myocardial preconditioning in dogs. Circ Res.

[52] Tokube K, Kiyosue T, Arita M (1996). Openings of cardiac KATP channel by oxygen free radicals produced by xanthine oxidase reaction. Am J Physiol.

[53] Gross GJ, Fryer RM (1999). Sarcolemmal versus mitochondrial ATP-sensitive K^+^ channels and myocardial preconditioning. Circ Res.

[54] Jovanovic A, Jovanovic S, Lorenz E, Terzic A (1998). Recombinant cardiac ATP-sensitive K^+^ channel subunits confer resistance to chemical hypoxia-reoxygenation injury. Circulation.

[55] Marinovic J, Bosnjak ZJ, Stadnicka A (2006). Distinct roles for sarcolemmal and mitochondrial adenosine triphosphate-sensitive potassium channels in isoflurane-induced protection against oxidative stress. Anesthesiology.

[56] Gumina RJ, Pucar D, Bast P (2003). Knockout of Kir6.2 negates
ischemic preconditioning-induced protection of myocardial
energetics. Am J Physiol Heart Circ Physiol.

[57] Marinovic J, Ljubkovic M, Stadnicka A (2008). Role of sarcolemmal ATP-sensitive potassium channel in oxidative stress-induced apoptosis: mitochondrial connection. Am J Physiol Heart Circ Physiol.

[58] Matejikova J, Kucharska J, Pinterova M (2009). Protection against ischemia-induced ventricular arrhythmias and myocardial dysfunction conferred by preconditioning in the rat heart: involvement of mitochondrial K(ATP) channels and reactive oxygen species. Physiol Res.

[59] Forbes RA, Steenbergen C, Murphy E (2001). Diazoxide-induced cardioprotection requires signaling through a redox-sensitive mechanism. Circ Res.

[60] Das B, Sarkar C (2006). Similarities between ischemic preconditioning and 17 beta-estradiol mediated cardiomyocyte KATP channel activation leading to cardioprotective and antiarrhythmic effects during ischemia/reperfusion in the intact rabbit heart. J Cardiovasc Pharmacol.

[61] Facundo HT, de Paula JG, Kowaltowski AJ (2007). Mitochondrial ATP-sensitive K^+^ channels are redox-sensitive pathways that control reactive oxygen species production. Free Radic Biol Med.

[62] Fryer RM, Eells JT, Hsu AK (2000). Ischemic preconditioning in rats: role of mitochondrial K(ATP) channel in preservation of mitochondrial function. Am J Physiol Heart Circ Physiol.

[63] Burwell LS, Nadtochiy SM, Brookes PS (2009). Cardioprotection by metabolic shut-down and gradual wake-up. J Mol Cell Cardiol.

[64] Stanley WC, Recchia FA, Lopaschuk GD (2005). Myocardial substrate metabolism in the normal and failing heart. Physiol Rev.

[65] Kussmaul L, Hirst J (2006). The mechanism of superoxide production by NADH:ubiquinone oxidoreductase (complex I) from bovine heart mitochondria. Proc Natl Acad Sci U S A.

[66] Fontaine E, Bernardi P (1999). Progress on the mitochondrial permeability transition pore: regulation by complex I and ubiquinone analogs. J Bioenerg Biomembr.

[67] Burwell LS, Nadtochiy SM, Tompkins AJ (2006). Direct evidence for S-nitrosation of mitochondrial complex I. Biochem J.

[68] Brookes PS, Shiva S, Patel RP, Darley-Usmar VM (2002). Measurement of mitochondrial respiratory thresholds and the control of respiration by nitric oxide. Methods Enzymol.

[69] Wojtovich AP, Brookes PS (2008). The endogenous mitochondrial complex II inhibitor malonate regulates mitochondrial ATP-sensitive potassium channels: implications for ischemic preconditioning. Biochim Biophys Acta.

[70] Brunori M, Forte E, Arese M (2006). Nitric oxide and the respiratory enzyme. Biochim Biophys Acta.

[71] Nadtochiy SM, Baker PR, Freeman BA, Brookes PS (2009). Mitochondrial nitroalkene formation and mild uncoupling in ischaemic preconditioning: implications for cardioprotection. Cardiovasc Res.

[72] Batthyany C, Schopfer FJ, Baker PR (2006). Reversible post-translational modification of proteins by nitrated fatty acids *in vivo*. J Biol Chem.

[73] Yabe K, Nasa Y, Sato M (1997). Preconditioning preserves mitochondrial function and glycolytic flux during an early period of reperfusion in perfused rat hearts. Cardiovasc Res.

[74] Zuurbier CJ, Eerbeek O, Meijer AJ (2005). Ischemic preconditioning, insulin, and morphine all cause hexokinase redistribution. Am J Physiol Heart Circ Physiol.

[75] Sun L, Shukair S, Naik TJ (2008). Glucose phosphorylation and mitochondrial binding are required for the protective effects of hexokinases I and II. Mol Cell Biol.

[76] Kwak HJ, Park KM, Choi HE, Park HY (2009). Protective mechanisms of NO preconditioning against NO-induced apoptosis in H9c2 cells: role of PKC and COX-2. Free Radic Res.

[77] Bolli R, Shinmura K, Tang XL (2002). Discovery of a new function of cyclooxygenase (COX)-2: COX-2 is a cardioprotective protein that alleviates ischemia/reperfusion injury and mediates the late phase of preconditioning. Cardiovasc Res.

[78] Shinmura K, Tang XL, Wang Y (2000). Cyclooxygenase-2 mediates the cardioprotective effects of the late phase of ischemic preconditioning in conscious rabbits. Proc Natl Acad Sci U S A.

[79] Farber NE, Pieper GM, Thomas JP, Gross GJ (1988). Beneficial effects of iloprost in the stunned canine myocardium. Circ Res.

[80] Hide EJ, Thiemermann C (1996). Sulprostone-induced reduction of myocardial infarct size in the rabbit by activation of ATP-sensitive potassium channels. Br J Pharmacol.

[81] Baines CP, Goto M, Downey JM (1997). Oxygen radicals released during ischemic preconditioning contribute to cardioprotection in the rabbit myocardium. J Mol Cell Cardiol.

[82] Ping P, Takano H, Zhang J (1999). Isoform-selective activation of protein kinase C by nitric oxide in the heart of conscious rabbits: a signaling mechanism for both nitric oxide-induced and ischemia-induced preconditioning. Circ Res.

[83] Teng JC, Kay H, Chen Q (2008). Mechanisms related to the cardioprotective effects of protein kinase C epsilon (PKC epsilon) peptide activator or inhibitor in rat ischemia/reperfusion injury. Naunyn Schmiedebergs Arch Pharmacol.

[84] Malhotra A, Kang BP, Hashmi S, Meggs LG (2005). PKCepsilon inhibits the hyperglycemia-induced apoptosis signal in adult rat ventricular myocytes. Mol Cell Biochem.

[85] Tappia PS, Dent MR, Dhalla NS (2006). Oxidative stress and redox regulation of phospholipase D in myocardial disease. Free Radic Biol Med.

[86] Asemu G, Dent MR, Singal T (2005). Differential changes in phospholipase D and phosphatidate phosphohydrolase activities in ischemia-reperfusion of rat heart. Arch Biochem Biophys.

[87] Raeburn CD, Zimmerman MA, Arya J (2002). Ischemic preconditioning: fact or fantasy?. J Card Surg.

[88] Arner ES, Holmgren A (2000). Physiological functions of thioredoxin and thioredoxin reductase. Eur J Biochem.

[89] Prieto-Alamo MJ, Jurado J, Gallardo-Madueno R (2000). Transcriptional regulation of glutaredoxin and thioredoxin pathways and related enzymes in response to oxidative stress. J Biol Chem.

[90] Turoczi T, Chang VW, Engelman RM (2003). Thioredoxin redox signaling in the ischemic heart: an insight with transgenic mice overexpressing Trx1. J Mol Cell Cardiol.

[91] Das DK, Maulik N (2003). Preconditioning potentiates redox signaling and converts death signal into survival signal. Arch Biochem Biophys.

[92] van Deel ED, Lu Z, Xu X (2008). Extracellular superoxide dismutase protects the heart against oxidative stress and hypertrophy after myocardial infarction. Free Radic Biol Med.

[93] Yamashita N, Hoshida S, Taniguchi N (1998). A "second window of protection" occurs 24 h after ischemic preconditioning in the rat heart. J Mol Cell Cardiol.

[94] Maczewski M, Duda M, Pawlak W, Beresewicz A (2004). Endothelial protection from reperfusion injury by ischemic preconditioning and diazoxide involves a SOD-like anti-O2- mechanism. J Physiol Pharmacol.

[95] McLeod CJ, Aziz A, Hoyt RF (2005). Uncoupling proteins 2 and 3 function in concert to augment tolerance to cardiac ischemia. J Biol Chem.

[96] Krauss S, Zhang CY, Scorrano L (2003). Superoxide-mediated activation of uncoupling protein 2 causes pancreatic beta cell dysfunction. J Clin Invest.

[97] Krauss S, Zhang CY, Lowell BB (2005). The mitochondrial uncoupling-protein homologues. Nat Rev Mol Cell Biol.

[98] Turrens JF, Thornton J, Barnard ML (1992). Protection from reperfusion injury by preconditioning hearts does not involve increased antioxidant defenses. Am J Physiol.

[99] Eguchi M, Monden K, Miwa N (2003). Role of MAPK phosphorylation in cytoprotection by pro-vitamin C against oxidative stress-induced injuries in cultured cardiomyoblasts and perfused rat heart. J Cell Biochem.

[100] Tsukaguchi H, Tokui T, Mackenzie B (1999). A family of mammalian Na^+^-dependent L-ascorbic acid transporters. Nature.

[101] Fujiwara M, Nagao N, Monden K (1997). Enhanced protection against peroxidation-induced mortality of aortic endothelial cells by ascorbic acid-2-O-phosphate abundantly accumulated in the cell as the dephosphorylated form. Free Radic Res.

[102] Arduini A, Mezzetti A, Porreca E (1988). Effect of ischemia and reperfusion on antioxidant enzymes and mitochondrial inner membrane proteins in perfused rat heart. Biochim Biophys Acta.

[103] Ferrari R, Ceconi C, Curello S (1985). Oxygen-mediated myocardial damage during ischemia and reperfusion: role of the cellular defenses against oxygen toxicity. J Mol Cell Cardiol.

[104] Porreca E, Del Boccio G, Lapenna D (1994). Myocardial antioxidant defense mechanisms: time related changes after reperfusion of the ischemic rat heart. Free Radic Res.

[105] Coudray C, Pucheu S, Boucher F (1992). Ischemia and reperfusion injury in isolated rat heart: effect of reperfusion duration on xanthine oxidase, lipid peroxidation, and enzyme antioxidant systems in myocardium. Basic Res Cardiol.

[106] Vanden Hoek T, Becker LB, Shao ZH (2000). Preconditioning in cardiomyocytes protects by attenuating oxidant stress at reperfusion. Circ Res.

[107] Blasig IE, Ebert B, Hennig C (1990). Inverse relationship between ESR spin trapping of oxyradicals and degree of functional recovery during myocardial reperfusion in isolated working rat heart. Cardiovasc Res.

[108] Costa VM, Silva R, Ferreira R (2009). Adrenaline in pro-oxidant conditions elicits intracellular survival pathways in isolated rat cardiomyocytes. Toxicology.

[109] Lee JW, Bobst EV, Wang YG (2000). Increased endogenous ascorbyl free radical formation with singlet oxygen scavengers in reperfusion injury: an EPR and functional recovery study in rat hearts. Cell Mol Biol (Noisy-le-grand).

[110] Ilangovan G, Zweier JL, Kuppusamy P (2004). Mechanism of oxygen-induced EPR line broadening in lithium phthalocyanine microcrystals. J Magn Reson.

[111] Zhu X, Liu B, Zhou S (2007). Ischemic preconditioning prevents in vivo hyperoxygenation in postischemic myocardium with preservation of mitochondrial oxygen consumption. Am J Physiol Heart Circ Physiol.

[112] Zhao X, He G, Chen YR (2005). Endothelium-derived nitric oxide regulates postischemic myocardial oxygenation and oxygen consumption by modulation of mitochondrial electron transport. Circulation.

[113] Liu S, Liu W, Ding W (2006). Electron paramagnetic resonance-guided normobaric hyperoxia treatment protects the brain by maintaining penumbral oxygenation in a rat model of transient focal cerebral ischemia. J Cereb Blood Flow Metab.

[114] Singhal AB, Wang X, Sumii T (2002). Effects of normobaric hyperoxia in a rat model of focal cerebral ischemia-reperfusion. J Cereb Blood Flow Metab.

[115] Colantuono G, Tiravanti EA, Di Venosa N (2008). Hyperoxia confers myocardial protection in mechanically ventilated rats through the generation of free radicals and opening of mitochondrial ATP-sensitive potassium channels. Clin Exp Pharmacol Physiol.

[116] Choi H, Kim SH, Chun YS (2006). *In vivo* hyperoxic preconditioning prevents myocardial infarction by expressing bcl-2. Exp Biol Med (Maywood).

[117] Sterling DL, Thornton JD, Swafford A (1993). Hyperbaric oxygen limits infarct size in ischemic rabbit myocardium *in vivo*. Circulation.

[118] Masini E, Salvemini D, Ndisang JF (1999). Cardioprotective activity of endogenous and exogenous nitric oxide on ischaemia reperfusion injury in isolated guinea pig hearts. Inflamm Res.

[119] Wink DA, Hanbauer I, Krishna MC (1993). Nitric oxide protects against cellular damage and cytotoxicity from reactive oxygen species. Proc Natl Acad Sci U S A.

[120] Rickover O, Zinman T, Kaplan D, Shainberg A (2008). Exogenous nitric oxide triggers classic ischemic preconditioning by preventing intracellular Ca^2+^ overload in cardiomyocytes. Cell Calcium.

[121] Shiva S, Sack MN, Greer JJ (2007). Nitrite augments tolerance to ischemia/reperfusion injury via the modulation of mitochondrial electron transfer. J Exp Med.

[122] Hill BG, Darley-Usmar VM (2008). S-nitrosation and thiol switching in the mitochondrion: a new paradigm for cardioprotection in ischaemic preconditioning. Biochem J.

[123] Dahm CC, Moore K, Murphy MP (2006). Persistent S-nitrosation of complex I and other mitochondrial membrane proteins by S-nitrosothiols but not nitric oxide or peroxynitrite: implications for the interaction of nitric oxide with mitochondria. J Biol Chem.

[124] Rossig L, Fichtlscherer B, Breitschopf K (1999). Nitric oxide inhibits caspase-3 by S-nitrosation *in vivo*. J Biol Chem.

[125] Cabigas BP, Su J, Hutchins W (2006). Hyperoxic and hyperbaric-induced cardioprotection: role of nitric oxide synthase 3. Cardiovasc Res.

[126] Ii M, Nishimura H, Iwakura A (2005). Endothelial progenitor cells are rapidly recruited to myocardium and mediate protective effect of ischemic preconditioning *via* "imported" nitric oxide synthase activity. Circulation.

[127] Takahashi S, Mendelsohn ME (2003). Synergistic activation of endothelial nitric-oxide synthase (eNOS) by HSP90 and Akt: calcium-independent eNOS activation involves formation of an HSP90-Akt-CaM-bound eNOS complex. J Biol Chem.

[128] Fontana J, Fulton D, Chen Y (2002). Domain mapping studies reveal that the M domain of hsp90 serves as a molecular scaffold to regulate Akt-dependent phosphorylation of endothelial nitric oxide synthase and NO release. Circ Res.

[129] Venema RC, Venema VJ, Ju H (2003). Novel complexes of guanylate cyclase with heat shock protein 90 and nitric oxide synthase. Am J Physiol Heart Circ Physiol.

[130] Shi Y, Baker JE, Zhang C (2002). Chronic hypoxia increases endothelial nitric oxide synthase generation of nitric oxide by increasing heat shock protein 90 association and serine phosphorylation. Circ Res.

[131] Muscari C, Bonafe F, Gamberini C (2004). Early preconditioning prevents the loss of endothelial nitric oxide synthase and enhances its activity in the ischemic/reperfused rat heart. Life Sci.

